# Well-differentiated liposarcoma of the spermatic cord: A case report

**DOI:** 10.1016/j.eucr.2021.101587

**Published:** 2021-01-29

**Authors:** Yuhei Shiba, Kenji Tamura, Yosuke Fukiishi, Shingo Ashida, Takashi Karashima, Keiji Inoue

**Affiliations:** Department of Urology, Kochi University, Nankoku, Japan

## Abstract

A 51-year-old man visited our hospital for voluntary cancer screening. Positron emission tomography and computed tomography (PET-CT) incidentally found a mass in the left scrotum. The patient was referred to our department. Magnetic resonance imaging suggested a diagnosis of a fat-containing tumor of the spermatic cord and excluded an inguinal hernia. High orchiectomy was performed due to a diagnosis of lipoma or liposarcoma. Histopathological diagnosis was well-differentiated liposarcoma. He has survived 28 months after surgery without recurrence. Spermatic cord tumors are difficult to diagnose with imaging, and attention must be paid to the possibility of malignant tumors.

## Introduction

Soft tissue sarcoma represents approximately 1% of all newly diagnosed tumors. Liposarcoma is the most common soft tissue sarcoma, accounting for 9.8–18%. Liposarcoma is a tumor that occurs mainly in adults, and its onset peaks between the ages of 40 and 60 years, with a slightly higher incidence in men. It most often occurs in the extremities, followed by the retroperitoneum. The incidence in the scrotum is 3.6%, which is a relatively rare disease. The origin of intrascrotal liposarcoma includes the spermatic cord (76%), testicular tunics (20%), and epididymis (4%), but in most cases, identification of the origin is difficult due to the size of the tumor and the degree of adhesion. Magnetic resonance imaging (MRI) provides good information for the precise localization of the tumor, but it cannot completely identify the type of tumor. Positron emission tomography and computed tomography (PET-CT) is also useful in cases of recurrence. In our case, the histopathological diagnosis was well-differentiated liposarcoma.

### Case presentation

**Fig. 1:**
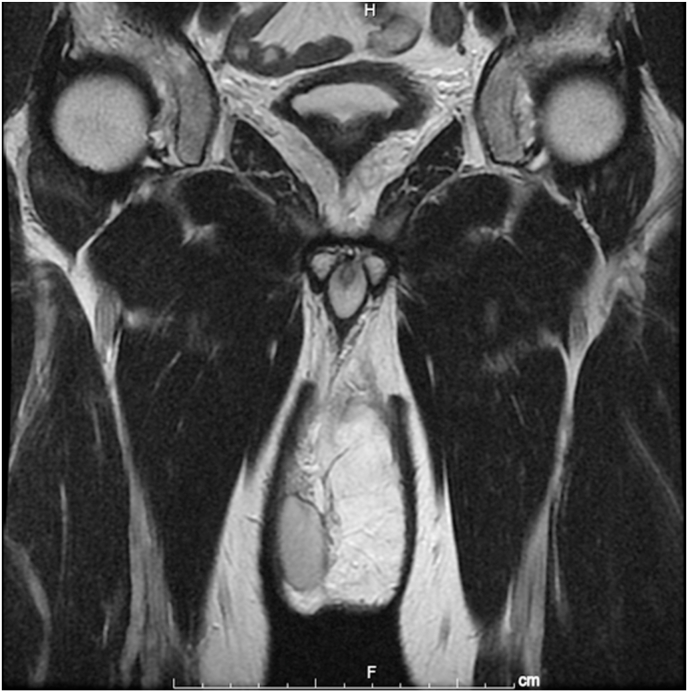
Pelvis MRI: T2-weighted coronal image.

**Fig. 2:**
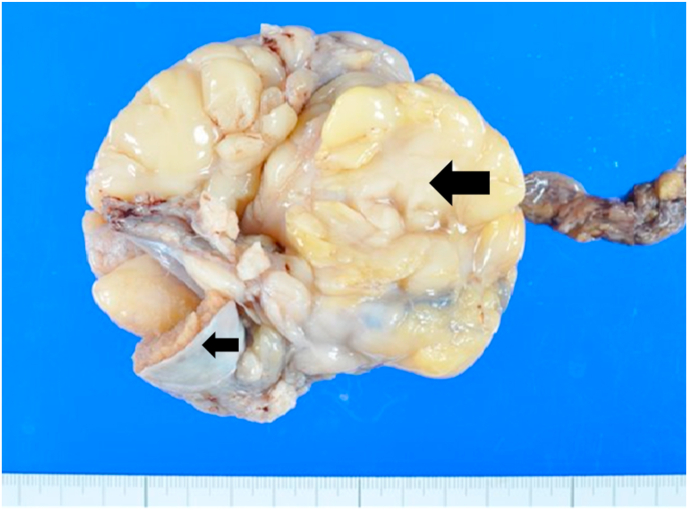
Macroscopic findings of the surgical specimen. (large arrow: tumor tissue, small arrow: normal testis).

**Fig. 3:**
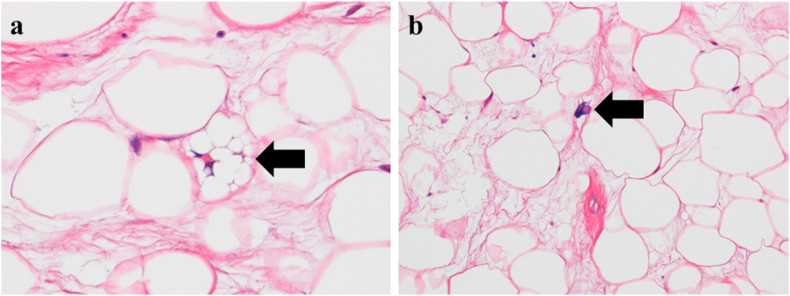
Histopathological examination revealed a well-differentiated liposarcoma of the spermatic cord. a) arrow: some lipoblasts (hematoxylin-eosin staining; magnification, × 400) and b) arrow: atypical stromal cells (hematoxylin-eosin staining; magnification, × 200).

The use of PET-CT for voluntary cancer screening of asymptomatic individuals is becoming common in Japan, although the utility of such screening is still controversial. A 51-year-old man visited our hospital for cancer screening with PET-CT, which incidentally found a mass in the left scrotum. He had been aware of painless swelling of the scrotum for 4 months, but he did not have this condition investigated. He attributed this swelling to the left hydrocele testis. He was introduced to our department for further investigation. Physical examination revealed a hard mass in the left scrotum that was larger than the size of a fist. MRI of the pelvis and scrotum revealed a fat-containing tumor in the spermatic cord (Fig. 1). Testicular tumor markers such as lactate dehydrogenase, β-human chorionic gonadotropin, and α-fetoprotein were normal, and no metastatic tumors were present. High orchiectomy was performed due to a diagnosis of lipoma or liposarcoma. The size of the excised specimen was 122 × 61 × 60 mm, and the macroscopic cut surface showed a yellow mass of soft tissue that partially touched the induration (Fig. 2). He was discharged 4 days after surgery. Histopathological examination revealed well-differentiated liposarcoma in the spermatic cord and no malignant tumor in the testis or epididymis (Fig. 3). The resection margin was negative. The patient was followed up without postoperative adjuvant therapy. No signs of local recurrence or distant metastasis were present 28 months after the operation.

## Discussion

The World Health Organization classification is used for the histopathological classification of liposarcoma as follows: 1) well-differentiated liposarcoma, 2) mucinous liposarcoma, 3) polymorphic liposarcoma, and 4) dedifferentiated liposarcoma. The prognosis of liposarcoma depends on the histology. Well-differentiated liposarcoma and mucinous liposarcoma have a better prognosis than other histological subtypes of liposarcoma, with 5-year survival rates of 85%, 77%, and 20%, respectively.^1^ Even with a well-differentiated liposarcoma, the prognosis is extremely poor when dedifferentiation is present.^2^ Dedifferentiated liposarcoma is defined as a malignant neoplasm that changes from a well-differentiated liposarcoma to a non-liposarcoma. Dedifferentiation occurs in a time-dependent manner in 10–15% of well-differentiated types. The average period until dedifferentiation is 7.7 years, and the 5-year survival rate after dedifferentiation is as low as 28%. Dedifferentiation often occurs after recurrent metastases.^3^ A recent study showed that well-differentiated liposarcoma expresses high levels of tissue inhibitor of metalloproteinase-4 (TIMP-4) and shows suppression of expression of yes-associated protein (YAP) and its paralog transcriptional coactivator with a PDZ-binding motif (TAZ), whereas dedifferentiated liposarcoma expresses high levels of tissue inhibitor of metalloproteinase-1 (TIMP-1) and activation of YAP/TAZ.^4^ The development of new diagnostic and therapeutic methods for liposarcoma that target YAP/TAZ signaling pathways controlled by TIMP-1 and TIMP-4 is expected.

Surgery is the first choice for treatment of spermatic liposarcoma. Some reports have shown that radiation therapy and chemotherapy are useful as adjuvant therapy, but in general, the effective rate is low, and no established treatment is available. Performing a high orchiectomy including removal of the surrounding normal tissue is important to obtain a negative margin. Tumor resection alone is often not sufficient because of local recurrence, which is also involved in the development of distant metastases that affect prognosis.^5^ Most metastases are hematogenous metastases, and the significance of standard lymph node dissection has not been established. Some reports have described recurrence more than 10 years after the operation, and long-term careful follow-up is required.

## Conclusion

Even in well-differentiated liposarcoma, the prognosis deteriorates significantly when dedifferentiated. Therefore, repeat surgery is necessary to obtain a favorable long-term prognosis if indications of local recurrence are present.

## Financial conflict of interest

None.

## Declaration of competing interest

None.
